# Cross-Protective Efficacy of Influenza Virus M2e Containing Virus-Like Particles Is Superior to Hemagglutinin Vaccines and Variable Depending on the Genetic Backgrounds of Mice

**DOI:** 10.3389/fimmu.2017.01730

**Published:** 2017-12-08

**Authors:** Yu-Jin Kim, Young-Tae Lee, Min-Chul Kim, Yu-Na Lee, Ki-Hye Kim, Eun-Ju Ko, Jae-Min Song, Sang-Moo Kang

**Affiliations:** ^1^Center for Inflammation, Immunity and Infection, Institute for Biomedical Sciences, Georgia State University, Atlanta, GA, United States; ^2^Animal and Plant Quarantine Agency, Gimcheon, South Korea; ^3^Department of Global Medical Science, Sungshin Women’s University, Seoul, South Korea

**Keywords:** influenza virus, M2e virus-like particles, BALB/C, C57BL/6, CD4 and CD8 T cells

## Abstract

Influenza virus M2 extracellular domain (M2e) has been a target for developing cross-protective vaccines. However, the efficacy and immune correlates of M2e vaccination are poorly understood in the different host genetic backgrounds in comparison with influenza vaccines. We previously reported the cross-protective efficacy of virus-like particle (M2e5x VLP) vaccines containing heterologous tandem M2e repeats (M2e5x) derived from human, swine, and avian influenza viruses. In this study to gain better understanding of cross-protective influenza vaccines, we compared immunogenicity and efficacy of M2e5x VLP, H5 hemagglutinin VLP (HA VLP), and inactivated H3N2 virus (H3N2i) in wild-type strains of BALB/c and C57BL/6 mice, and CD4 and CD8 knockout (KO) mice. M2e5x VLP was superior to HA VLP in conferring cross-protection whereas H3N2i inactivated virus vaccine provided high efficacy of homologous protection. After M2e5x VLP vaccination and challenge, BALB/c mice induced higher IgG responses, lower lung viral loads, and less body weight loss when compared with those in C57BL/6 mice. M2e5x VLP but not H3N2i immune mice after primary challenges developed strong immunity against a secondary heterosubtypic virus as a model of future pandemics. M2e5x VLP and HA VLP vaccines were able to raise IgG isotypes in CD4 KO mice. T cells were found to contribute to cross-protection by playing a role in reducing lung viral loads. In conclusion, M2e5x VLP vaccination induced better cross-protection than HA VLP, and its efficacy varied depending on the genetic backgrounds of mice, supporting the important roles of T cells.

## Introduction

Influenza A viruses are divided into different subtypes based on hemagglutinin (HA) and neuraminidase (NA) proteins on the surface of the virus. At present, antigenically different influenza viruses with 1 combination out of 18 HA and 11 NA subtype molecules are known to exist and continue to mutate in diverse hosts including humans, birds, and pigs (1, 2). Vaccination has been considered as the most effective measure to protect against influenza virus infection causing severe respiratory and pulmonary disease. Current influenza vaccination based on HA immunity is effective in conferring protection against vaccine HA strain-matched and closely related influenza viruses. However, the efficacy of current influenza vaccination based on highly changeable HA is low or non-protective when the circulating viruses are mutated or a new pandemic virus emerges. Therefore, the development of broadly cross-protective vaccines is of a high priority to improve the efficacy of vaccination and to prevent future pandemic outbreaks.

The extracellular domain of M2 (M2e) is well conserved among influenza A viruses (3–5). M2 itself is a poor immunogen. M2e as a cross-protective antigenic target has been reported using a variety of carrier vehicles such as hepatitis B virus core particles (6, 7), human papillomavirus L proteins (8), phage Qβ-derived protein cores (9), keyhole limpet hemocyanin (10), bacterial outer membrane complexes (11), liposomes (12), cholera toxin subunit (13), and flagellin (14). Also, different adjuvants were used in the M2e vaccines, which include Freund’s adjuvant (15), monophosphoryl lipid A (12, 13), cholera toxin subunits (16, 17), and heat-labile endotoxin (7, 18). Our previous studies demonstrated that virus-like particle (VLP) vaccines presenting heterologous tandem repeat M2e (M2e5x VLP) were effective in inducing cross-protection against different subtypes of influenza A viruses in the absence of adjuvants (19). Most of previous M2e-based vaccine studies have been carried out in BALB/c mice known to be a high responder (20, 21). No IgG antibodies and T cell responses specific for M2e were induced in C57BL/6 mice that were primed with M2 DNA and boosted with M2 recombinant adenovirus (21). We reported that M2e5x VLP could raise IgG antibodies but provide low efficacy of cross-protection in C57BL/6 mice without adjuvants (22).

Unlike seasonal outbreaks, influenza pandemics can occur when a completely new influenza virus strain emerges. These kinds of viruses can be generated directly from animal reservoirs or the result of genetic reassortments in previously circulating viruses (23), emphasizing the needs of developing broadly cross-protective vaccines (24). In addition, vaccines should be effective in genetically diverse populations. In this study to compare different influenza vaccine platforms, we first determined the induction of IgG isotype antibodies and efficacy of protection in BALB/c and C57BL/6 mice after vaccination with M2e5x VLP, H5 VLP containing H5 subtype HA, or whole inactivated H3N2 virus (H3N2i). We also determined the impact of immunization with different vaccine platforms after primary challenges on developing immunity against the secondary challenge with an antigenically different virus, as a model of pandemics in future. Finally, the roles of CD4 and CD8 T cells in inducing IgG antibodies and protective efficacy were investigated after vaccination of CD4 knockout (CD4 KO) and CD8 knockout (CD8 KO) mice during primary and secondary infection.

## Materials and Methods

### Viruses and Vaccines

A/Philippines/2/1982 (H3N2), A/California/04/2009 (H1N1), and reassortant H5N1 (rgH5N1) containing H5 HA with polybasic residues removed from H5N1 A/Indonesia/05/2005 and NA and six internal genes from A/PR/8/1934 were propagated in embryonated hen’s eggs as previously described (25, 26). To prepare whole inactivated H3N2 vaccine (H3N2i), A/Philippines/2/1982 (H3N2) virus was treated with formalin at a final concentration of 1:4,000 (v/v) as described previously (27). M2e5x VLP containing tandem repeat of heterologous M2e derived from human (2xM2e), swine (1x), and avian (2xM2e) influenza virus was prepared as detailed in previous study (19). H5 VLP presenting H5 type of HA protein from A/Indonesia/05/2005 was previously described (26). Briefly, Sf9 insect cells were co-infected with recombinant baculoviruses expressing influenza M1 matrix core protein and M2e5x or H5 HA. M2e5x VLP and H5 VLP vaccines were purified from cell culture supernatants containing released VLP by sucrose gradient ultracentrifugation. The contents of M2e epitopes (~6%) and HA (~9%) incorporated onto VLPs were confirmed by enzyme-linked immunosorbent assay (ELISA) and western blots (19, 26).

### Immunization and Challenge

Adult wild-type and mutant mice (6–10 weeks old) used in this study include BALB/c, C57BL/6, CD4KO (B6.129S2-*Cd4^tm1Mak^*/J), and CD8KO mice (B6.129S2-*Cd8a^tm1Mak^*/J), and were obtained from the Jackson Laboratory (Sacramento, CA, USA). Groups of each strain of mice (*n* = 10, males and females) were intramuscularly (i.m.) immunized with 10 µg (total proteins) of M2e5x VLP, H5 HA VLP (H5 HA from A/Vietnam/1203/2004), or H3N2i (whole inactivated A/Philippines/2/1982 virus) by prime–boost regimen at a 3-week interval. At 4 weeks after boost immunization, immunized mice were then challenged intranasally with a sublethal dose of A/Philippines/2/1982 H3N2 (0.8× LD_50_) or rgH5N1 (0.8× LD_50_). For secondary challenge, mice that survived primary infection were challenged with a lethal dose of A/California/04/2009 H1N1 (10× LD_50_) at 7 weeks after the primary infection. Survival rate and body weight loss were daily monitored for 14 days upon infection. All animal experimental procedures in this study were approved by the Georgia State University Institutional Animal Care and Use Committee review boards. Infected mice that reached 25% body weight loss as an endpoint were euthanized and recorded as dead.

### Determination of Antibody Responses

Influenza virus-specific or M2e-specific antibody levels were determined by ELISA. Immune sera were serially diluted and then applied to the 96-well plates (Corning Incorporated, Tewksbury, MA, USA) that were coated with M2e peptide, inactivated A/Indonesia rgH5N1, or inactivated A/Philippines/2/1982 H3N2 virus as previously described (28, 29). IgG and IgG isotype levels were determined by HRP conjugated anti-mouse IgG, IgG1, IgG2a, IgG2b, or IgG2c (SouthernBiotech, Birmingham, AL, USA) and tetramethylbenzidine (eBioscience, San Diego, CA, USA) as a substrate (30).

### Lung Virus Titers

Lung samples were collected from the groups of mice at 7 days after challenge. Viral titers were determined as described previously (19). Briefly, lung extracts were serially diluted in 10-fold and injected into 10 days old embryonated chicken eggs. The 50% of egg infectious dose (EID_50_) was calculated by the Reed–Muench method.

### Hemagglutination Inhibition (HI) Assay

Hemagglutination inhibition assay was performed as previously described (31). Immune sera were mixed with cholera filtrates as the receptor destroying enzyme purchased from Sigma Aldrich (St. Louis, MO, USA) and then incubated at 37°C. At 16 h after incubation, samples were heat inactivated at 56°C for 30 min. Serially twofold diluted sera were incubated with eight HA units of A/Philippines/2/1982 (H3N2) or rgH5N1 for 30 min, followed by adding 0.5% chicken red blood cells (Lampire Biological Laboratories, Pipersville, PA, USA) to determine HI titers.

### Cytokine ELISPOT

To detect interferon (IFN)-γ and interleukin (IL)-4 spot-forming cells, splenocytes (5 × 10^5^ cells/well) and lung cells (2 × 10^5^ cells/well) were cultured on 96-well plates coated with anti-mouse IFN-γ or IL-4 monoclonal antibodies (BD Biosciences, San Diego, CA, USA) in the presence of M2e peptide (4 µg/ml). The cytokine spots were developed with biotinylated mouse IFN-γ, IL-4 antibodies, and alkaline phosphatase labeled streptavidin (BD Pharmingen, San Diego, CA, USA). The spots were visualized with a 3,3′-diaminobenzidine substrate and counted by an ELISpot reader (BioSys, Miami, FL, USA).

### *In Vivo* Protection Assay of Immune Sera

To test whether M2e5x VLP-immune sera contribute to cross-protection, *in vivo* protection assay was performed as described previously (19). Briefly, heat inactivated sera at 56°C for 30 min were diluted and mixed with a lethal dose (2× LD_50_) of A/Philippines/2/1982 (H3N2). Naïve mice were intranasally infected with a mixture (50 µl) of virus and sera, and the survival rates and body weight changes were daily monitored for 14 days.

### Intracellular Cytokine Staining and Flow Cytometry Assay

For intracellular cytokine analysis, harvested cells were stimulated with M2e peptides and then stained with fluorescence-labeled anti-mouse CD4 and anti-mouse CD8 antibodies. Subsequently, the cells were made permeable by using the Cytofix/Citoperm kit (BD Biosciences, San Diego, CA, USA) and intracellular cytokines were stained with anti-mouse IFNγ and anti-mouse Granzyme B antibodies. All antibodies were purchased from eBioscience. Stained cells were analyzed using LSR Fortessa (BD Biosciences, San Diego, CA, USA) and FlowJo software (Tree Star).

### Statistical Analysis

All results are expressed as the mean ± SEM. Significant differences among treatments were evaluated by two-way ANOVA. *p*-Values of less than or equal to 0.05 were considered statistically significant.

## Results

### C57BL/6 Mice Display Lower Levels of M2e-Specific IgG Responses after M2e5x VLP Vaccination than BALB/c Mice

**Figure 1:**
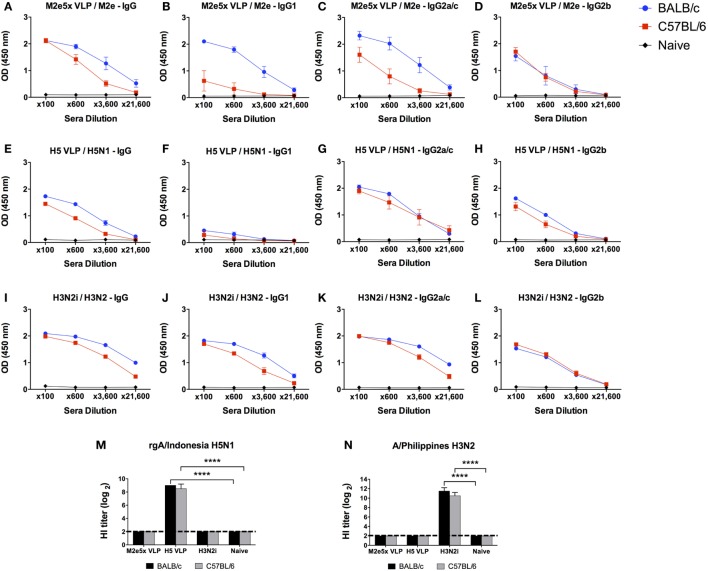
IgG isotype antibody responses in BALB/c and C57BL/6 mice after vaccination with different influenza vaccines. Each group of mice (*n* = 10) was prime and boost intramuscularly immunized with 10 µg of M2e5x virus-like particle (VLP) **(A–D)**, H5 hemagglutinin VLP (HA VLP) **(E–H)**, or inactivated A/Philippines virus (H3N2i) **(I–L)**. Antibody levels of IgG **(A,E,I)**, IgG1 **(B,F,J)**, IgG2a in BALB/c mice or IgG2c in C57BL/6 mice **(C,G,K)**, and IgG2b **(D,H,L)** were detected by enzyme-linked immunosorbent assay (ELISA). Sera were serially diluted and ELISA was performed by using vaccine-specific antigens which are M2e peptide, inactivated A/Indonesia (rgH5N1i), and H3N2i. Error bars indicates mean ± SEM. **(M,N)** Hemagglutination inhibition (HI) titers. HI titers against rgH5N1 **(M)** and H3N2 **(N)** were determined from immune sera of M2e5x VLP, H5 HA VLP, or H3N2i. Statistical significances were determined by 2-way ANOVA. ****p < 0.0001.

We determined IgG isotype antibody responses to different influenza vaccine platform antigens in BALB/c and C57BL/6 mice (Figure 1). Groups of mice were i.m. immunized with 10 µg of M2e5x VLP, H5 HA VLP, or H3N2i. IgG isotypes specific for different influenza virus antigens were determined at 2 weeks after boost immunization. The M2e5x VLP BALB/c mice showed substantially high levels of IgG1, IgG2a, and IgG2b antibodies specific for M2e (Figures 1A–D). The H5 HA VLP BALB/c mice developed high levels of IgG2a and IgG2b antibodies specific for the homologous rgH5N1 virus antigen. The level of IgG1 antibodies specific rgH5N1 virus was significantly low in both BALB/c and C57BL/6 mice (Figures 1E–H). The H3N2i group in BALB/c mice raised significant levels of IgG1, IgG2a, and IgG2b antibodies specific for the same H3N2 vaccine virus (Figures 1I–L). H5 HA VLP and H3N2i immune sera showed HA inhibition activity against each antigen-specific rgH5N1 and A/Philippines/2/82 (H3N2) virus, respectively (Figures 1M,N).

C57BL/6 mice immunized with M2e5x VLP also developed IgG2c and IgG2b isotype antibodies binding to M2e antigens but at lower levels than BALB/c mice (Figures 1A–D). Whereas, comparably high levels of IgG2b antibodies specific for rgH5N1 and H3N2i virus antigens were induced in the H5 HA VLP and H3N2i C57BL/6 groups (Figures 1E–L).

Overall, both BALB/c and C57BL/6 mice developed differential levels of IgG isotypes depending on the types of antigens and vaccine platforms. M2e5x VLP induced higher IgG1 isotype antibody responses than H5 HA VLP. Both BALB/c and C57BL/6 mice that were immunized with H3N2i vaccine developed IgG and class-switched IgG antibody responses at similarly high levels. H5 HA VLP and M2e5x VLP vaccines showed a tendency of inducing IgG2c isotype antibodies. Meanwhile, H3N2i immunization induced similar levels of class-switched IgG antibodies in both BALB/c and C57BL/6 mice.

### M2e5x VLP Is More Effective in Conferring Cross-Protection than H5 VLP

**Figure 2:**
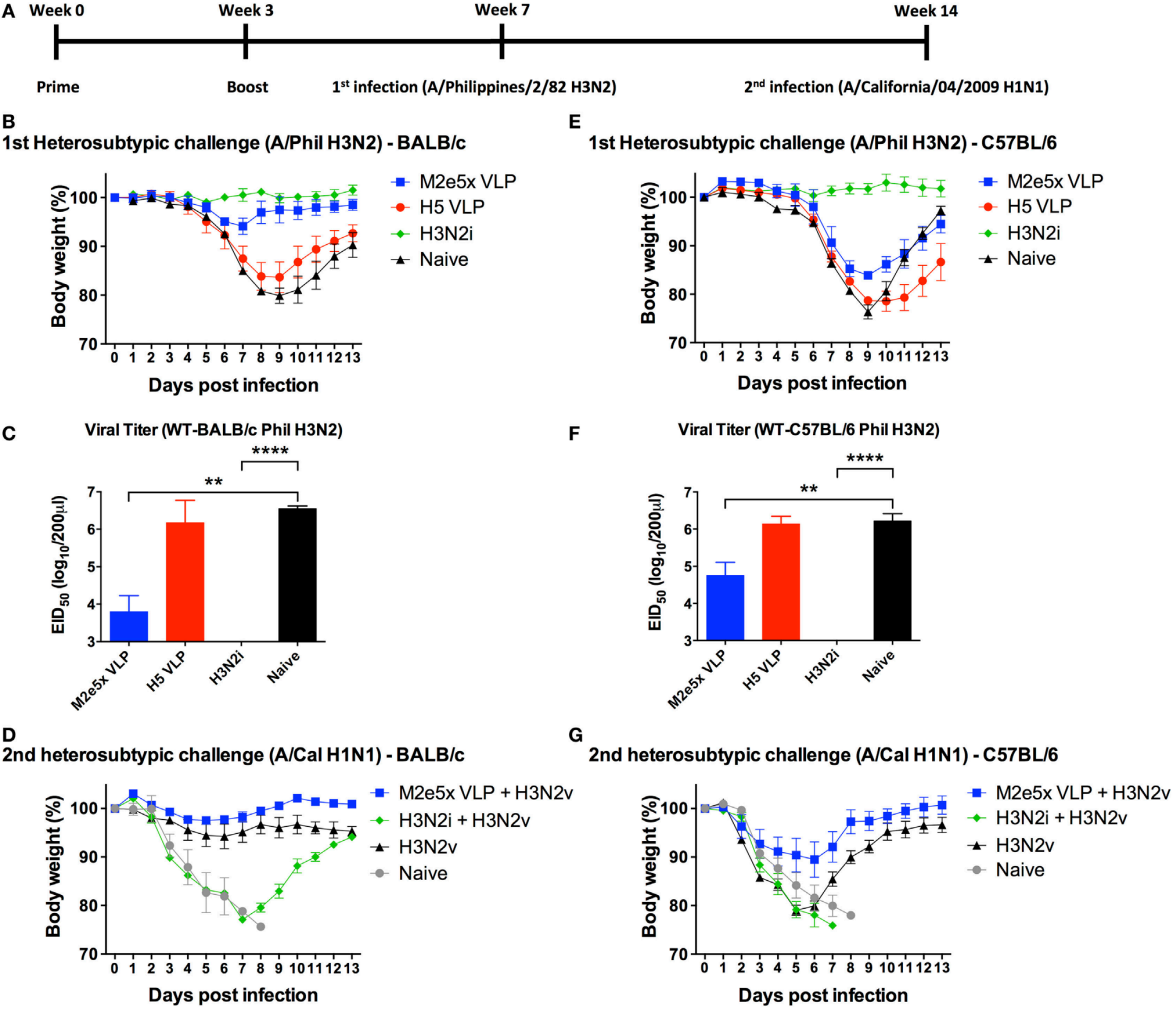
Differential efficacy of protection by M2e5x virus-like particle (VLP), H5 VLP, or H3N2i after primary H3N2 virus and secondary H1N1 virus challenge in BALB/c and C57BL/6 mice. **(A)** Time schedule for experiments of vaccination, first challenge infection with H3N2 virus, and second challenge infection with H1N1 virus. BALB/c **(B,C)** and C57BL/6 **(E,F)** mice were challenged with A/Philippines/2/82 (H3N2) (H3N2v) at 4 weeks after boost with M2e5x VLP, H5 hemagglutinin VLP (HA VLP), or H3N2i. Body weights were monitored for 14 days **(B,E)**. Lung viral titers were determined by the egg inoculation assay **(C,F)**. The detection limit of 50% of egg infectious dose (EID_50_) was 1.7 Log10. At 7 weeks after first infection with A/Philippines/2/82/(H3N2), BALB/c **(D)**, and C57BL/6 **(G)** mice were challenged with a different subtype of influenza virus (A/California/04/2009 H1N1) and body weight changes were monitored for 14 days. H3N2 infection only group was the naïve infection group of **(B,E)**. Data represent the mean ± SEM. Statistical significances were evaluated by two-way ANOVA. **p < 0.01, ****p < 0.0001.

To determine cross-protective efficacy, naïve and vaccinated mice were challenged with H3N2 (A/Philippines/82) virus (Figure 2A). As expected, H3N2i vaccination induced complete protection against the homologous H3N2 virus in both BALB/c and C57BL/6 mice (Figures 2B,E). Also, H5 HA VLP immunization induced protection against homologous rgH5N1 virus (data not shown). In contrast, H5 HA VLP vaccination did not induce protection against heterosubtypic H3N2 virus as evidenced by severe body weight loss in BALB/c and C57BL/6 mice similar to naïve control (Figures 2B,E). BALB/c mice immunized with M2e5x VLP showed protection against H3N2 virus challenge despite a slight weight loss (Figure 2B). C57BL/6 mice immunized with M2e5x VLP showed significant weight loss (15–18%) compared with the same vaccination of BALB/c mice (Figure 2E). Nonetheless, the M2e5x VLP-immunized group of C57BL/6 mice showed less weight loss indicating better cross-protection than the naïve control or H5 HA VLP group (Figure 2E).

Protective efficacy was further confirmed by lung viral titers at day 7 after H3N2 virus challenge (Figures 2C,F). The naïve and H5 HA VLP groups showed the highest lung viral titers in a range of 10^6^–10^7^ EID_50_ (per lung per milliliter) in contrast to the H3N2i group with lung viral clearance below the detection limit. The M2e5x VLP group exhibited approximately 100- and 20-folds lower lung viral titers compared with the H5 HA VLP group in BALB/c and C57BL/6 mice, respectively. Overall, the results of lung viral titers show a correlation with protection as indicated by weight loss.

### M2e5x VLP-Immunized Mice That Survived Primary Infection Develop Immunity against Antigenically Different Virus Infection As a Model of Future Pandemic

Outbreaks of pandemics are unpredictable and current vaccination is not effective in preventing pandemics. To address this critical issue, the vaccinated mice that survived the H3N2 virus primary challenge were exposed 7 weeks later to the secondary infection with a lethal dose of heterosubtypic H1N1 virus (A/California/07/2009) as a model of future pandemic. H3N2i vaccination did not provide protection against the secondary H1N1 virus, as shown by severe weight loss (over 22% in BALB/c mice) and no survivals (0%) in C57BL/6 mice (Figures 2D,G). The M2e5x VLP group exhibited the best protection and recovery against the secondary H1N1 virus infection in both BALB/c and C57BL/6 mice (Figures 2D,G). BALB/c mice with M2e5x VLP vaccination showed better protection against secondary heterosubtypic virus challenge than the corresponding C57BL/6 mouse group. In a similar trend, naïve BALB/c mice that survived primary H3N2 infection were protected against secondary H1N1 2009 virus (Figure 2D) whereas naïve C57BL/6 mice surviving primary H3N2 infection were not well protected against secondary H1N1 2009 virus (Figure 2G). Taken together, BALB/c mice with vaccination or primary H3N2 virus infection showed stronger immunity during the secondary heterosubtypic H1N1 virus infection than C57BL/6 mice, suggesting mouse strain differences in protective efficacy by virus infection or vaccination.

### BALB/c Mice Induce Higher Levels of M2e-Specific T Cell Responses than C57BL/6 Mice

**Figure 3:**
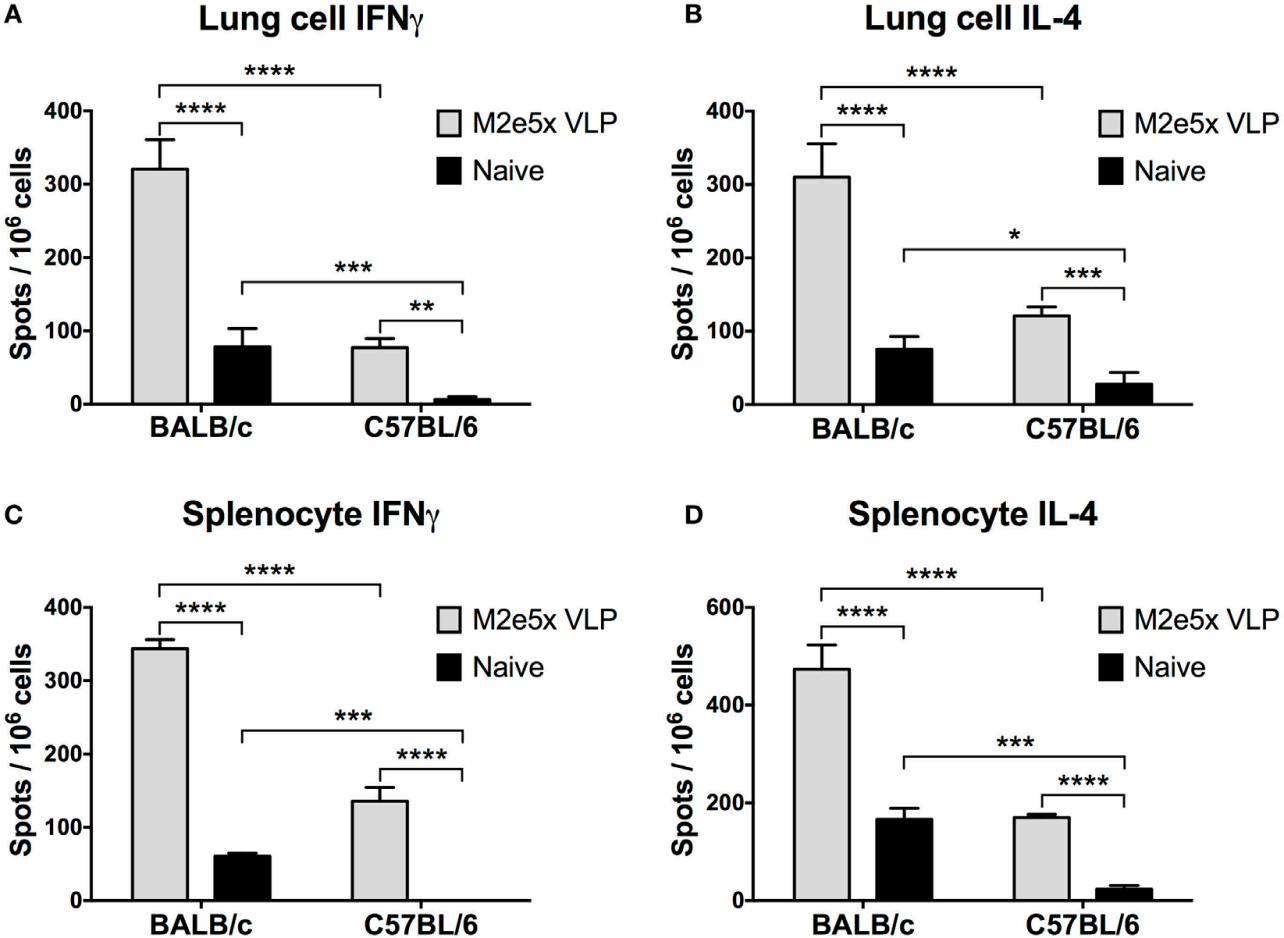
Cytokine-producing T cell responses induced by M2e5x virus-like particle (VLP) immunization in C57BL/6 and BALB/c mice after challenge. **(A)** Interferon (IFN)-γ-secreting cells in lungs. **(B)** Interleukin (IL)-4-secreting cells in lungs. **(C)** IFN-γ-secreting cells in splenocytes. **(D)** IL-4-secreting cells in splenocytes. Lung cells and splenocytes were isolated from C57BL/6 and BALB/c mice previously immunized with M2e5x VLP at day 7 post-challenge (A/Philippines/2/82 H3N2). Cytokine-producing cell spots were counted by ELISPOT reader. Data represent the mean ± SEM. Statistical significances were evaluated by two-way ANOVA. *p < 0.05, **p < 0.01, ***p < 0.001, ****p < 0.0001.

To better understand a difference in the cross-protective efficacy between C57BL/6 and BALB/c mice, we determined cellular immune responses by measuring the levels of IFN-γ and IL-4 cytokines secreted into culture supernatants after *in vitro* stimulation with M2e peptides (Figure 3). M2e5x VLP-immunized BALB/c mice showed significantly higher levels of IFN-γ and IL-4 cytokine secreting cells both in the lung and the spleen cells collected day 7 post-H3N2 challenge compared with M2e5x VLP-immunized C57BL/6 mice (Figure 3). These results indicate the possibility that a genetic background of BALB/c mice is more effective in inducing M2e-specific or virus-specific T cell responses than that of C57BL/6 mice.

**Figure 4:**
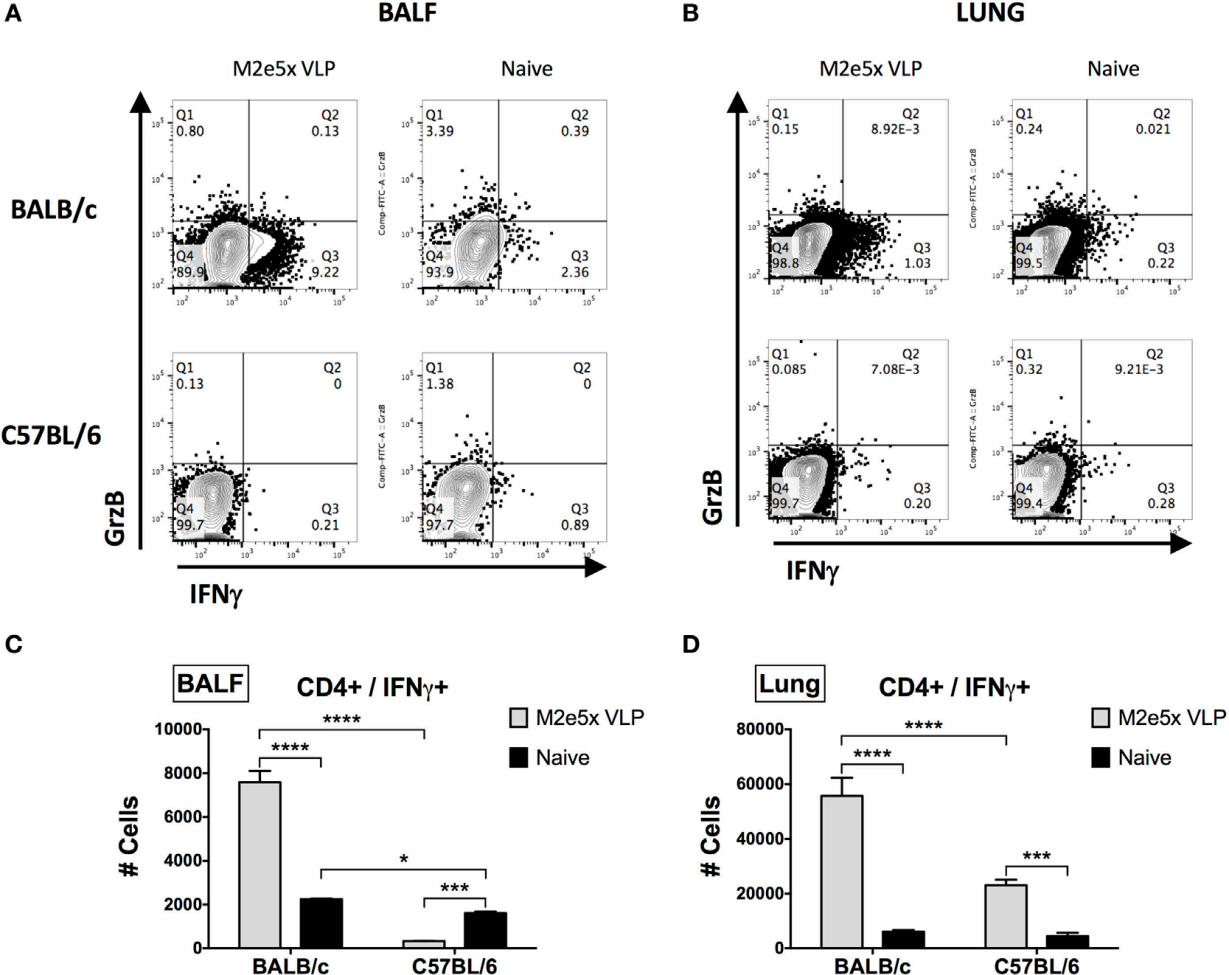
Intracellular cytokine staining of CD4^+^ T cells under the condition of M2e5x virus-like particle (VLP) immunization in C57BL/6 and BALB/c mice after challenge. **(A,B)** Representative flow cytometry profiles of interferon (IFN)-γ- and granzyme B-secreting CD4^+^ T cells in bronchoalveolar lavage fluids (BALF) **(A)** and lungs **(B)**. **(C)** The cellularity of IFN-γ-secreting CD4^+^ T cells in BALF. **(D)** The cellularity of IFN-γ-secreting CD4^+^ T cells in lungs. The numbers of cells were indicated as the number per mouse **(C,D)**. After gating CD4^+^ cells, IFN-γ^+^ or granzyme B^+^ cells were measured by flow cytometry of intracellularly stained cells. # Cells indicate the numbers in BALF and lungs collected per mouse **(C,D)**. Data represent the mean ± SEM. Statistical significances were evaluated by two-way ANOVA. *p < 0.05, ***p < 0.001, ****p < 0.0001.

To further confirm the M2e-specific T cell responses, we carried out intracellular staining and flow cytometry assays for quantification of CD4 and CD8 T cells secreting IFN-γ and granzyme B in lungs and bronchoalveolar lavage fluids (BALF) at 7 days after viral challenge. Significantly high levels of IFN-γ in CD4 T cells were detected in M2e5x VLP-immunized BALB/c mice (Figure 4). In contrast, C57BL/6 mice showed much lower levels of IFN-γ secreting CD4 T cells in lungs and BALF than BALB/c mice (Figure 4). IFN-γ secreting lung CD4 T cells induced by M2e5x VLP vaccination and virus challenge were observed at a higher level compared with that in naïve mice after infection (Figures 4B,D). In BALF, M2e5x VLP-immunized C57BL/6 mice showed a lower number of IFN-γ secreting CD4 T cells than the naïve mouse control (Figures 4A,C). Although the reason for this odd finding is not clear in C57BL/6 mice, this might be due to the delayed recruitment of the effector CD4 T cells to the airway area as a result of better control of lung viral loads. In lungs, C57BL/6 mice immunized with M2e5x VLP showed significantly higher IFN-γ secreting CD4 T cells than the naïve mouse control (Figure 4D) in a similar pattern with the case in BALB/c mice. The combined IFN-γ secreting CD4 T cells in BALF and lungs were significantly higher in M2e5x VLP-immunized C57BL/6 mice (Figures 4C,D). M2e5x VLP vaccination did not increase IFN-γ secretion from antigen-specific CD8 T cells (Figure S1 in Supplementary Material). However, BALB/c mice induced significantly higher levels of granzyme B-secreting CD8 T cells than C57BL/6 mice at day 7 after viral infection (Figure S1 in Supplementary Material). In summary, BALB/c mice have a genetic background resulting in higher T cell responses to M2e5x VLP vaccination and influenza viral infection.

### M2e5x VLP and H5 VLP Vaccines Induce IgG Isotype-Switched Antibodies in CD4 KO Mice at Different Levels

**Figure 5:**
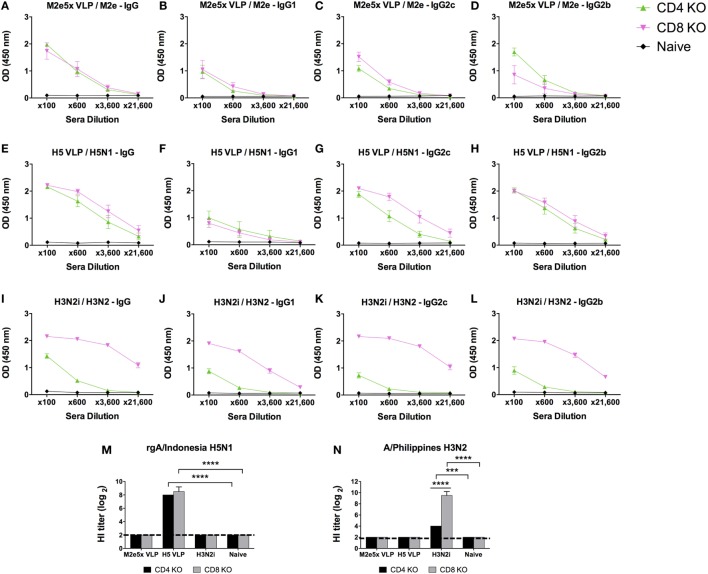
IgG isotype antibody responses in CD4 knockout (CD4 KO) and CD8 knockout (CD8 KO) mice after vaccination with different influenza vaccines. Each group of CD4 KO and CD8 KO mice (*n* = 10) was prime and boost intramuscularly immunized with 10 µg of M2e5x virus-like particle (VLP) **(A–D)**, H5 VLP **(E–H)**, or H3N2i **(I–L)**. Antibody levels of IgG **(A,E,I)**, IgG1 **(B,F,J)**, IgG2c **(C,G,K)**, and IgG2b **(D,H,L)** were detected by enzyme-linked immunosorbent assay (ELISA). The same influenza antigens were used for ELISA as described in Figure 2. Error bars indicates mean ± SEM. **(M,N)** Hemagglutination inhibition (HI) titers. HI titers against rgH5N1 **(M)** and H3N2 **(N)** were determined from immune sera of M2e5x VLP, H5 VLP, or H3N2i. Statistical significances as determined by 2-way ANOVA: ***p < 0.001, ****p < 0.0001.

CD4 T cells are known to play critical roles in inducing isotype-switched IgG antibodies (32). To determine the roles of T cells in inducing IgG isotype-switched antibodies and protective immunity after influenza vaccination, we immunized CD4 KO and CD8 KO mice (Figure 5). Vaccine dose and immunization regimens were the same as described in wild-type mice above. CD4 KO mice immunized with M2e5x VLP induced significant levels of M2e-specific IgG antibodies (Figures 5A–D). CD8 KO mice immunized with M2e5x VLP developed similar levels of M2e-specific IgG antibodies (Figures 5A–D). H5 VLP immunization was able to induce rgH5N1 virus-specific IgG antibodies in a similar trend with M2e5x VLP in both T cell KO mice (Figures 5E–H). CD4 KO mice immunized with H3N2i induced significantly lower levels of H3N2 virus-specific IgG, IgG2c, and IgG2b isotype antibodies (Figures 5I–L) than those in wild-type C57BL/6 mice (Figures 1I–L). However, H3N2i immunized CD8 KO mice developed similar levels of H3N2 virus-specific IgG antibodies with wild-type mice (Figures 5I–L).

### T Cells Contribute to Cross-Protection by M2e5x VLP Vaccine

**Figure 6:**
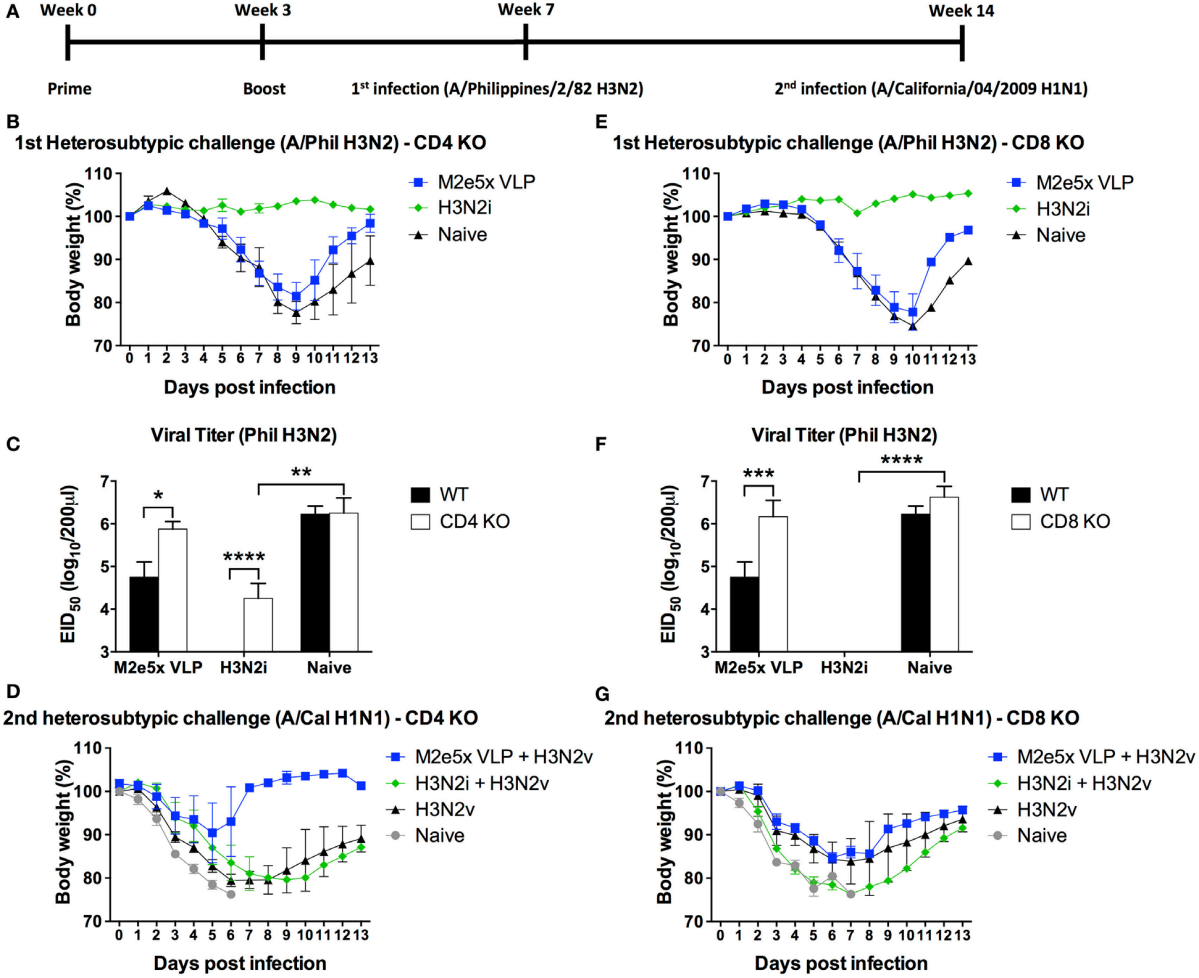
Efficacy of protection in CD4 knockout (CD4 KO) and CD8 knockout (CD8 KO) mice by different influenza vaccines after primary H3N2 virus and secondary H1N1 virus challenge. **(A)** Time schedule for experiments of vaccination, first challenge infection with H3N2 virus, and second challenge infection with H1N1 virus. CD4 KO **(B–D)** and CD8 KO **(E–G)** mice were challenged with A/Philippines/2/82 (H3N2) (H3N2v) at 4 weeks after boost with M2e5x virus-like particle (VLP) or H3N2i. Body weights were monitored for 14 days. Lung viral titers were determined by the egg inoculation assay **(C,F)**. The detection limit of 50% of egg infectious dose (EID_50_) was 1.7 Log10. At 7 weeks after first infection with A/Philippines/2/82/(H3N2), CD4 KO **(D)** and CD8 KO **(G)** mice were challenged with a different subtype of influenza virus (A/California/04/2009 H1N1) and body weight changes were monitored for 14 days. H3N2 virus infection only group was the naïve infection group of **(B,E)**. Data represent the mean ± SEM. Statistical significances were evaluated by two-way ANOVA. *p < 0.05, **p < 0.01, ***p < 0.001, ****p < 0.0001.

The roles of CD4 and CD8 T cells in conferring vaccine-induced protection were determined using KO mouse models (CD4 KO and CD8 KO) after vaccination and challenge (Figure 6). M2e5x VLP vaccinated CD4 KO mice displayed a progressive weight loss to a similar degree as in naïve CD4 KO mice but showed a better recovery at later time points (Figure 6B). A high-lung viral titer at day 7 after challenge was observed in the M2e5x VLP CD4KO group, which is similar to the one observed in naïve infection (Figure 6C), suggesting a role of CD4 T cells in M2e-immune mediated cross-protection. H3N2i vaccination of CD4 KO induced protection against homologous virus challenge although there was a substantial lung viral titer in CD4 KO mice (Figure 6C). H3N2i immune sera from CD4 KO mice significantly inhibited the HA activity of homologous virus (Figures 5M,N). These results suggest that CD4 T cells play a role in preventing severe weight loss and clearing lung viral loads by M2e5x VLP vaccination. As expected from high levels of IgG antibodies, H3N2i vaccinated CD8 KO mice showed no weight loss after homologous H3N2 virus challenge (Figure 6E) similar to the one induced in C57BL/6 mice (Figure 2E). M2e5x VLP vaccinated CD8 KO mice showed similar infection symptoms and high-lung viral loads similar to naïve CD8 KO mice against H3N2 virus challenge (Figures 6E,F). Thus, CD8 T cells also play an important role in preventing severe weight loss and in clearing lung viral loads in M2e5x VLP-immunized mice.

CD4 knockout (CD4 KO) mice with M2e5x VLP vaccination showed a moderate level of protection against secondary heterosubtypic virus challenge (Figure 6D) in a similar pattern with the corresponding C57BL/6 mouse groups (Figure 2G). CD8 KO mice immunized with M2e5x VLP showed more severe body weight loss compared with CD4 KO and wild-type mice (Figure 6G). Taken together, M2e5x VLP-mediated immunity is partially dependent on CD8 T cells during the secondary heterosubtypic virus infection whereas primary infection-mediated immunity is related with both CD4 and CD8 T cells.

### M2-Specific Immune Sera Play a Role in Conferring Cross-Protection

**Figure 7:**
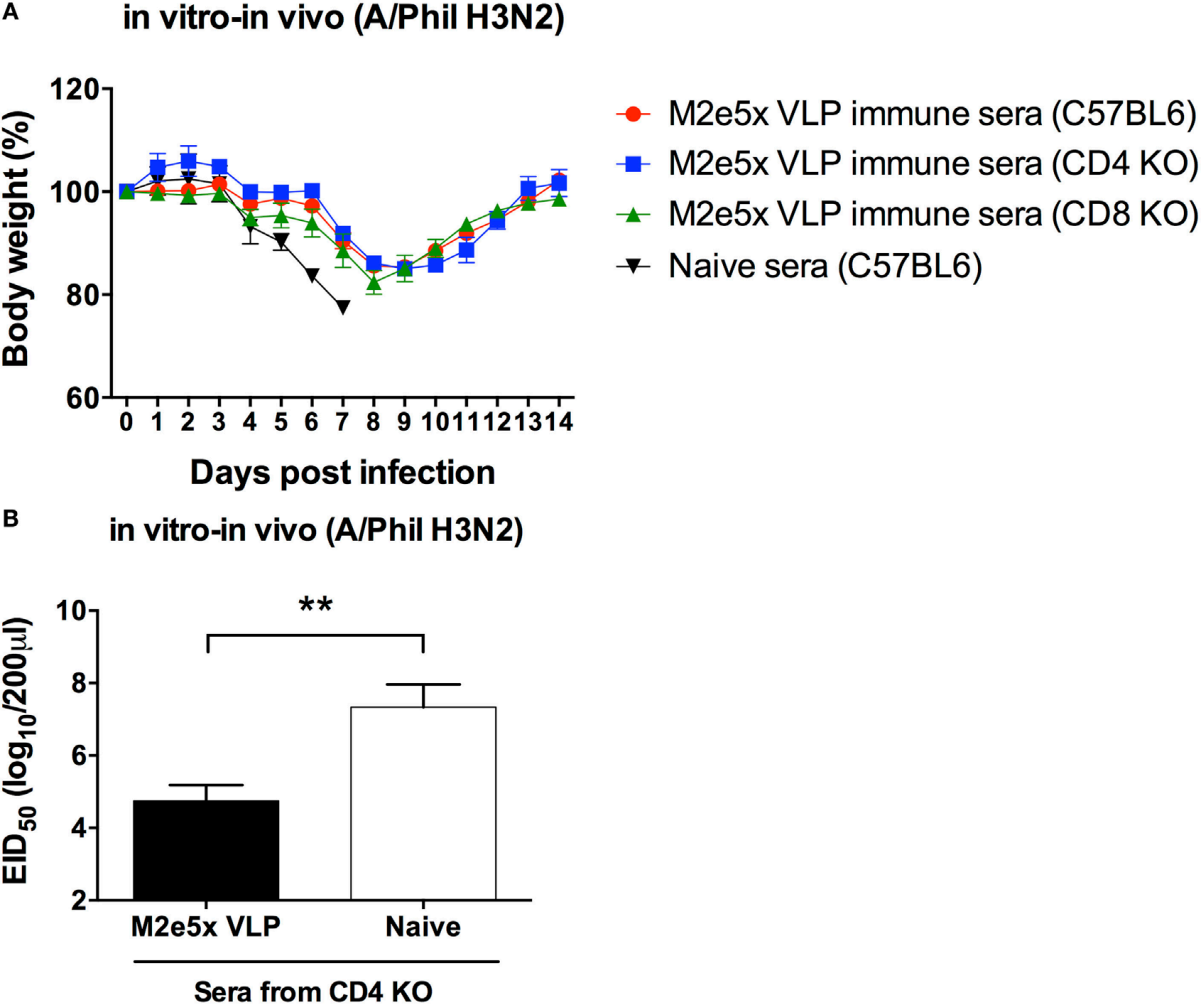
M2e antibodies in T cell-deficient mice show similar capacity to confer protection in naïve mice. Immune sera collected from immunized C57BL/6, CD4 knockout (CD4 KO), and CD8 knockout (CD8 KO) mice were incubated with influenza virus (A/Philippines/2/82 H3N2). Naive mice were intranasally infected with a mixture of a lethal dose of influenza virus and immune sera or naïve sera. Body weights were monitored for 14 days **(A)**. Lung viral titers day 7 post-infection was determined by the egg inoculation assay **(B)**. The detection limit of 50% of egg infectious dose (EID_50_) was 1.7 Log10. Statistical significances were analyzed by 2-way ANOVA. **p < 0.01.

M2e5x VLP was found to induce significant levels of M2e-specific antibodies in C57BL/6, CD4 KO, and CD8 KO mice. Thus, we determined the roles of M2e-immune sera in conferring protection independent of T cell immunity as detailed in Section “Materials and Methods.” Naïve mice were infected with a mixture of H3N2 virus and immune sera collected from different genotypic naïve mice or M2e5x VLP-immunized mice, and then weight changes and survival rates were daily monitored (Figure 7A). M2e5x VLP-immune sera from CD4 KO and CD8 KO mice were found to contribute to protection against H3N2 virus at a similar level as observed in M2e5x VLP-immune sera from C57BL/6 mice. Consistent with body weight monitoring results, M2e5x VLP-immune sera from CD4 KO mice representatively showed significantly lower levels of lung viral titers than naïve control sera (Figure 7B). Taken together, these results suggest that M2e-immune sera from different strains and genotypes of mice confer similar levels of protection regardless of genetic backgrounds and T cell immunity, preventing severe weight loss, and increasing survival rates and recovery.

## Discussion

Most subunit vaccines including cross-protective influenza A virus M2 vaccines have been investigated in BALB/c mice (19, 33–38). A limited set of influenza M2 vaccine studies has been performed in C57BL/6 mice (21, 22). In our previous studies (19, 35), we have developed an M2e targeting M2e5x VLP vaccine and studied cross-protective efficacy mostly in BALB/c mice. In this study of comparing HA-based H5 VLP and inactivated whole influenza virus (H3N2i) vaccines, we investigated heterosubtypic cross-protective efficacies of M2e5x VLP in mice with different genetic backgrounds and the roles of T cells in inducing IgG antibodies and protection using KO mutant mouse models. M2e5x VLP vaccination induced higher levels of IgG antibodies and protective efficacy in BALB/c mice than that in C57BL/6 mice. The efficacy of cross-protection by M2e5x VLP was higher than that by H5 VLP but lower compared with homologous protection by H3N2i immunization. M2e5x VLP vaccinated mice that were protected against primary challenge with H3N2 virus have developed future immunity to secondary infection against H1N1 virus, which was not induced in H3N2i immunized mice. M2e5x VLP and H5 VLP vaccines were able to raise substantial amounts of isotype-switched IgG antibodies in CD4 KO mice although antibody levels were lower than those in CD8 KO and C57BL/6 mice. In addition to immune sera containing M2e-specific antibodies, both CD4 and CD8 T cells were found to play roles in clearing lung viral loads and in better recovery after M2e5x VLP vaccination and virus challenge.

Efficacy studies in inbred mouse strains might not be predictive in genetically diverse human populations. A previous study reported that no IgG antibodies specific for M2e were induced in C57BL/6 mice that were primed with M2 DNA and boosted with M2 recombinant adenovirus (21). Also, antibody responses and protection to HA DNA vaccination were reported to be lower in C57BL/6 mice than those in BALB/c mice (39). In this study, M2e5x VLP raised similar or lower levels of IgG, IgG2c, and IgG2b isotype antibodies in C57BL/6 mice compared with those in BALB/c mice. Whereas, H5 HA VLP and H3N2i vaccines developed similar levels of IgG isotype antibodies in C57BL/6 and BALB/c mice. M2e5x VLP and H5 HA VLP vaccines were immunogenic and able to induce IgG isotype antibodies even in CD4 KO mice, suggesting an alternative pathway of inducing CD4-independent IgG antibodies. H3N2i vaccine appears to require CD4 T helper cells for effective induction of IgG antibodies. Presenting HA proteins on the VLP platform was shown to induce Th1-type antibody responses and enhanced protection compared with soluble form HA protein (28) or split influenza vaccines (40). In line with these results, H1 HA VLP was more effective in developing IgG antibodies in CD4 KO mice compared with the same H1N1 strain inactivated split influenza virus vaccine (data not shown). VLP itself appears to grant immunogenic properties to the antigens on it. VLP vaccines were reported to stimulate dendritic cells *in vitro* and *in vivo* and to produce inflammatory cytokines (41). In addition, VLP-loaded dendritic cells stimulated the induction of T cell responses *in vitro* (42). These unique properties of VLP vaccines likely attribute to inducing Th1-type IgG antibodies in BLAB/c and C57BL/6 mice as well as in CD4 KO and CD8 KO mice.

Despite the result that M2e5x VLP had immunogenic properties of inducing IgG antibodies, the protective efficacy was variable in genetically different strains and mutant mice. M2e5x VLP showed higher cross-protection in BALB/c mice than that in C57BL/6 mice. The efficacy of M2e5xVLP in lowering lung viral replication in CD4 KO and CD8 KO mice was even lower compared with that in C57BL/6 mice. Although H3N2i vaccination induced protection preventing weight loss against homologous primary virus even in CD4 KO mice, H3N2i immune mice were not protective during heterosubtypic secondary virus infection at a later time despite significant viral replication during the first infection. In contrast, infection permissive M2e5x VLP vaccination after primary challenge developed sufficient immunity in BALB/c mice against secondary heterosubtypic virus, consistent with previous studies in BALB/c mice (25, 43, 44). C57BL/6 mice with M2e5x VLP showed lower efficacy of secondary immunity than BALB/c mice, but significantly higher efficacy than naïve C57BL/6 mice displaying high-viral loads during primary infection. High-viral replication during primary infection accompanying severe weight loss in naïve C57BL/6 mice was not effective in inducing immunity against secondary heterosubtypic virus infection. This aspect provides evidence of beneficial effects on developing future immunity by inducing cross-protective M2e-immunity as shown in M2e5x VLP-immunized C57BL/6 mice. The efficacy of future immunity against secondary virus infection was diminished in CD8 KO mice, suggesting that both CD8 T cells and M2e antibodies might be major immune correlates contributing to protection against heterosubtypic virus infection. In addition, this study provides evidence that high-viral replication in naïve C57BL/6 mice would not be sufficient for developing immunity against heterosubtypic virus probably due to less efficacy of inducing granzyme B-secreting CD8 T cell responses in C57BL/6 mice compared with those in BALB/c mice.

Several mechanisms have been reported for protection by M2e-immunity. Immune sera containing M2e antibodies regardless of strains of mice conferred similar levels of protection in naïve mice. Thus, M2e antibodies induced in C57BL/6 and CD4 KO mice may have similar capability of protection. Since lung viral loads were higher in CD4 KO and CD8 KO mice than those in C57BL/6 mice in the M2e5x VLP group, it is possible that both CD4 and CD8 T cells play a role of effector functions in lowering viral loads (45). Alternatively, CD4 T cells may help to sustain the cytotoxic T cell response. Other mechanisms for M2e-immune-mediate protection include Fc receptor (FcR) (29, 35, 46), FcR-mediated opsonophagocytosis by macrophages (33), and natural killer cells (34). It appears that FcR is a key mediator for conferring cross-protection by M2e antibodies. In addition, this study highlights the impacts of host genetic backgrounds and T cell responses in cross-protection.

## Author Contributions

Y-JK is the first author. S-MK and Y-JK led this project and were responsible for experimental design, data analysis, and writing this manuscript. Y-TL, M-CK, Y-NL, K-HK, and E-JK contributed to performing experiments and data analysis. J-MS contributed to generating the H5 HA VLP vaccine and challenge virus. S-MK is the corresponding author.

## Conflict of Interest Statement

The authors declare that the research was conducted in the absence of any commercial or financial relationships that could be construed as a potential conflict of interest.

## Supplementary Material

Figure S1 Intracellular cytokine staining of CD8^+^ T cells in C57BL/6 and BALB/c mice. Flow cytometry profiles of interferon (IFN)-γ- and granzyme B-secreting CD8^+^ T cells in bronchoalveolar lavage fluids (BALF) and lungs. After gating CD8^+^ cells, IFN-γ^+^ or granzyme B^+^ cells were measured by flow cytometry of intracellularly stained cells.
