# Supplementary figures and images for: Cross-Protective Efficacy of Influenza Virus M2e Containing Virus-Like Particles Is Superior to Hemagglutinin Vaccines and Variable Depending on the Genetic Backgrounds of Mice
Source: Front Immunol. 2017 Dec 8;8:1730. doi: 10.3389/fimmu.2017.01730 (PMC5727122; doi:10.3389/fimmu.2017.01730)

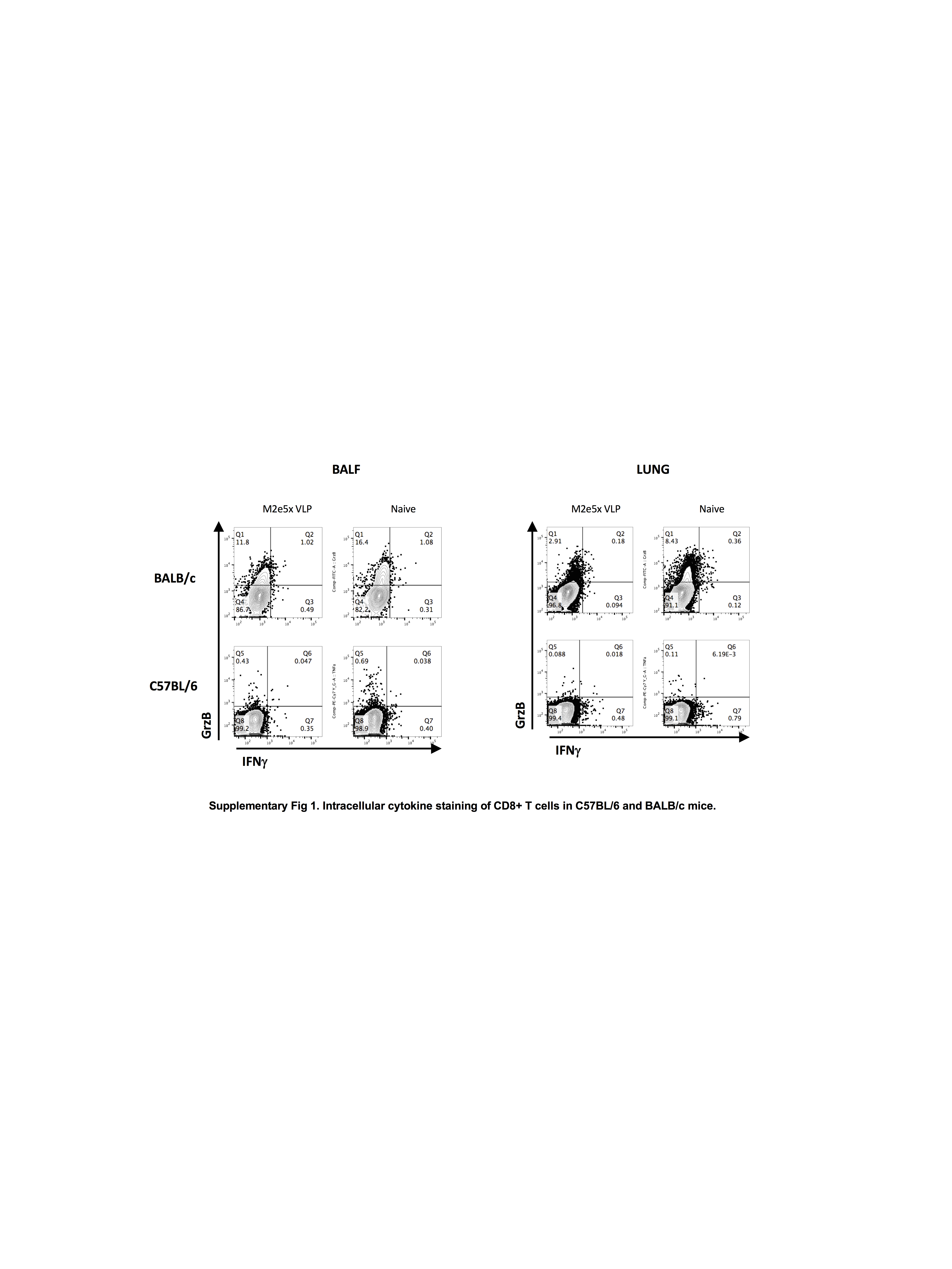

Supplement: Figure S1 — Intracellular cytokine staining of CD8+ T cells in C57BL/6 and BALB/c mice. Flow cytometry profiles of interferon (IFN)-γ- and granzyme B-secreting CD8+ T cells in bronchoalveolar lavage fluids (BALF) and lungs. After gating CD8+ cells, IFN-γ+ or granzyme B+ cells were measured by flow cytometry of intracellularly stained cells. [file image_1.jpg]
